# Aspects épidémiologique, clinique et anatomopathologique du mélanome CHU Yalgado Ouédraogo de Ouagadougou (Burkina Faso)

**DOI:** 10.11604/pamj.2015.20.220.6351

**Published:** 2015-03-11

**Authors:** Nina Korsaga-Somé, Nayi Zongo, Edgar Ouangré, Patrice Tapsoba, Muriel Sidnomam Ouédraogo, Léopold Ilboudo, Alban Bassolet, Maryam Sanou, Djounitana Djimtibaye, Fatou Barro-Traoré, Pascal Niamba, Adama Traoré

**Affiliations:** 1 Service de Dermatologie-Vénéréologie, CHU Yalgado Ouédraogo, Ouagadougou, Burkina Faso; 2 Service de Chirurgie Générale et Digestive, CHU Yalgado Ouédraogo, Ouagadougou, Burkina Faso

**Keywords:** Mélanome, Burkina Faso, tumeur maligne

## Abstract

Le mélanome est une tumeur maligne développée aux dépens des mélanocytes. Ses caractéristiques épidémiologiques restent peu étudiées dans les populations africaines. D'où le but de cette étude. Il s'est agit d'une étude rétrospective descriptive de janvier 2009 à décembre 2012 et prospective en 2013. Pour la partie rétrospective, nous avons inclus tous les patients atteints de mélanome confirmé par l'examen histopathologique et ayant un dossier clinique exploitable. Pour la partie prospective, nous avons inclus tous les cas de mélanomes recensés en 2013. Les variables étudiées étaient les caractéristiques sociodémographiques, anatomocliniques et thérapeutiques. Au total 19 patients étaient inclus, avec un âge médian de 53,42 ans et 10 patients de sexe féminin. La durée moyenne d’évolution de la maladie était de 3,7 ans. Les lésions étaient surtout ulcero-bourgeonnantes (13), hyperpigmentées (15), et siégeaient sur les plantes (14). Le type et acro-lentigineux était le plus fréquent (13). L'indice de Clarck a été mesuré dans 3 cas, l'indice de Breslow dans aucun cas. Les métastases étaient présentes chez 10 patients. Le traitement était surtout chirurgical. Les 11 patients inclus de manière retrospective étaient ont été perdus de vue. Un décès était noté parmi les 8 patients inclus de manière prospective. Le retard à la consultation semblait être le principal facteur de mauvais pronostic dans notre contexte. Une sensibilisation de la population et du personnel de santé sur les signes précoces de la maladie, permettrait probablement de réduire la morbidité.

## Introduction

Le mélanome est une tumeur maligne développée aux dépens des mélanocytes. Ces mélanocytes synthétisent la mélanine qui est obtenue par un mélange de quantité variable de deux sortes de pigments: les phaeomélanines et les eumélanines. Ces deux types de pigments synthétisés jouent un rôle différent. Les eumélanines sont des pigments de couleur noire ou brune, absorbant totalement la lumière et exerçant donc un réel pouvoir photoprotecteur (type de mélanine prédominant dans les peaux mates). Lors d'une irradiation prolongée, les eumélanines se regroupent au-dessus du noyau des kératinocytes afin de protéger le matériel génétique de la cellule. Les phaeomélanines (pigment prépondérant dans les peaux claires) sont des pigments jaunes orangés qui n'ont, au contraire, pas ou peu de rôle photoprotecteur et peuvent même générer des radicaux libres potentiellement mutagènes pour l'ADN. Le rôle de l'exposition solaire dans le mélanome est démontré, avec un risque plus élevé pour les expositions solaires intenses et intermittentes [1, 2], chez des individus de phototype clair et ayant facilement des coups de soleils. L´incidence du mélanome, à l’échelle planétaire, est très variable en fonction de la latitude (soleil) et des caractéristiques ethniques des populations. Cette incidence atteint des sommets (jusqu’à 60 nouveaux cas pour 100 000 habitants et par an) chez les blancs dans certaines régions d'Australie, alors qu'elle est très faible dans les pays où les sujets sont noirs ou asiatiques [3]. La mortalité est directement liée à l’épaisseur tumorale. Dans certains pays européens, des campagnes de détection précoce et le développement de filières d'accès plus rapides aux soins, ont permis de détecter des tumeurs moins épaisses, et de réduire la mortalité. Les caractéristiques épidémiologiques du mélanome dans les populations africaines restent peu étudiées en termes de fréquence, de mortalité, d'accès aux filières de soins et aux médicaments des stades métastatiques. Néanmoins, sa prévalence est plus faible, et la localisation aux paumes, aux plantes et aux muqueuses, qui sont les zones les moins pigmentées chez le noir est proportionnellement plus représentée. L'incidence annuelle serait de 0,4 pour 100 000 habitants en Afrique [4], elle n´est pas connue au Burkina Faso.

Les buts de notre étude étaient de déterminer: quelle était la prévalence hospitalière du mélanome au CHUYalgadoOuédraogo (CHU-YO) de Ouagadougou? Quelle était le profil sociodémographique de ces patients? Quelle en étaient les formes cliniques et histopathologiques, et le délai entre les premiers signes cliniques et la prise en charge? Et enfin, quelle était la prise en charge offerte à ces patients?

## Méthodes

**Cadre, période et type de l’étude:** l’étude a été réalisée dans les services de Dermatologie-Vénéréologie et de Chirurgie Générale et Digestive du CHU Yalagado Ouédraogo de Ouagadougou. Une première partie de l’étude était rétrospective sur la base des dossiers des patients consultants au CHU YO de janvier 2009 à décembre 2012. La deuxième partie était prospective de janvier 2013 à décembre 2013.

**Critères d'inclusion:** pour la partie rétrospective, nous avons inclus tous les patients atteints de melanoma confirmé par l'examen histopathologique et ayant un dossier clinique exploitable. Pour la partie prospective, nous avons procédé à un échantillonnage exhaustif de tous les cas de mélanomes recensés pendant l'année 2013.

**Identification des cas et collecte des données:** pour la période rétrospective, nous avons recherché dans les registres de consultation et d'hospitalisation tous les diagnostics de mélanome en notant le nom et le numéro du dossier des patients. Ensuite nous avons sorti tous les dossiers de cas de mélanome. Nous les avons examinés pour ne retenir que ceux qui étaient exploitables (ceux comportant les variables recherchées). Nous avons également recherché les compte-rendu histopathologiques, et nous avons renseigné les fiches de collecte à partir de ces dossiers. Les données ont été recueillies sur une fiche de collecte comportant des variables suivantes: les caractéristiques sociodémographiques des patients: âge, sexe, profession, milieu de résidence; les caractéristiques cliniques: durée d’évolution, signes fonctionnels, saignement, type et coloration de la lésion, siège, adénopathies et métastases; les caractéristiques histolopathologiques: type de mélanome, niveau de Clarck et de Breslow; les caractéristiques thérapeutiques: chirurgie d'exérèse, curage ganglionnaire, chimiothérapie, amputation. Les données ont été saisies et analysées avec le logiciel EPI-INFO dans sa version 3.5.1.

## Résultats

### Caractéristiques sociodémographiques

Nous avons colligé au total 19 cas de mélanomes dont 5 cas colligés dans le service de Dermatologie-Vénéréologie et 14 cas dans le service Chirurgie Générale et Digestive. Parmi ces 19 patients, 11 cas ont été inclus de manière retrospective et 8 cas de manière prospective, il n'y avait pas eu d'exclusion. Selon l'année, il y avait 2 cas de mélanome en 2009 comme en 2010, 3 cas en 2011, 4 cas en 2012 et 8 cas en 2013. En 2013, le service de Dermatologie-vénéréologie a enregistré 1600 nouvelles consultations et celui de Chirurgie viscérale et digestive 3635 nouveaux patients. Ce qui donnait pour l'année 2013 une incidence de 0,36% de mélanome. Dix de ces patients étaient de sexe féminin soit, un sex ratio de 0,9. Leur âge médian était de 53,42 ans avec des extrêmes de 23 et 80 ans. Le Tableau 1 donne la répartition des patients par tranches d’âge. Onze patients venaient du milieu rural, et selon le statut socioprofessionnel, 9 étaient des femmes au foyer, 6 des cultivateurs, 2 des commerçants et il y avait un étudiant et un fonctionnaire.

**Table 1:** Répartition des patients atteints de mélanome par tranches d’âge

Tranches d’âge/an	Effectif (n)
20-30	03
31-40	02
41-50	03
51-60	02
61-70	06
71-80	03
Total	19

### Caractéristiques cliniques et histopathologiques

**Figure 1:**
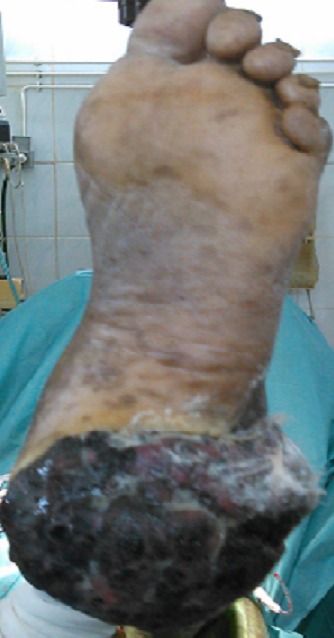
Mélanome plantaire: lésion ulcéro-bourgeonnante hyperpigmentée. Source: collection Service de Chirurgie générale et Digestive CHU-YO, Ouagadougou, Burkina Faso

**Figure 2:**
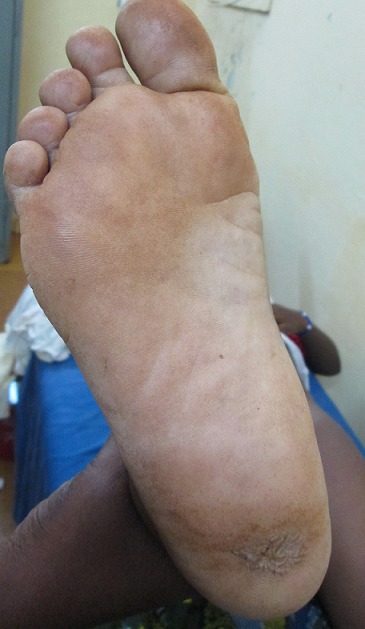
Mélanome plantaire achromique. Source: collection service de Chirurgie générale et Digestive CHU-YO, Ouagadougou, Burkina Faso

La durée moyenne d’évolution de la maladie avant la consultation était de 3,7 ans avec des extrêmes de 1 et 10 ans. Le Tableau 2 donne la répartition des patients selon la durée d’évolution de la maladie. Les lésions étaient douloureuses chez 12 patients et hémorragiques chez 10 patients. Des adénopathies inguinales étaient cliniquement décelables chez 6 patients. Les lésions se présentaient sous forme de macules hyperpigmentées dans 3 cas et de tumeurs dans 16 cas dont 13 lésions tumorales ulcero-bourgeonnantes (Figure 1). Ces lésions tumorales étaient hyperpigmentées dans 15 cas et achromique dans 1 cas (Figure 2). Les lésions siégeaient aux membres inferieurs dans 17 cas, dont 14 cas sur les plantes et 3 cas sur les talons. Les deux autres lésions étaient localisées au tronc. Le mélanome était acro-lentigineux dans 13 cas, nodulaire dans 3 cas et à extension superficielle dans 3 cas. L'indice de Clarck était mesuré dans 3 cas (II, IV et V). L'indice de Breslow n’était mesuré dans aucun cas. Les métastases étaient présentes chez 10 patients dont 6 cas de métastases ganglionnaires, un cas de métastase hépatique, un cas de métastase osseuse, un cas de métastase cérébrale et un cas de métastase pulmonaire. Le traitement a consisté en une chirurgie de propreté associée à une chimiothérapie chez 4 patients, une exérèse chirurgicale couplée à un curage ganglionnaire chez 6 patients et une exérèse chirurgicale seule chez 8 patients. Les 11 patients inclus de manière rétrospective étaient perdus de vue, ils n’étaient pas non plus joignables au téléphone (pronostic fatal?). Parmi les 8 patients inclus de manière prospective, nous avons noté un décès au 9ème mois de traitement.

**Table 2:** Répartition des patients atteints de mélanome en fonction de la durée d’évolution de la maladie

Durée d’évolution en année	Effectif (n)
< ou = 1	05
02	03
03	03
04	02
05	03
07	01
10	02
**Total**	**19**

## Discussion

Notre étude montrait que le mélanome dans notre région n’était pas exceptionnel, que les malades consultaient à un stade déjà avancé, avec un délai de plus de 3 ans entre les premiers signes constatés et la prise en charge. Ces mélanomes plantaires sont en effet interprétés souvent comme résultant de traumatisme et d'infections. Il y a donc un travail d’éducation spécifique à faire en direction des patients et des structures de soins primaires pour réduire ce délai. Les limites de notre travail étaient: d'abord le caractère rétrospectif avec les données manquantes dans les dossiers cliniques notamment pour la prise en charge et le suivi après l'exérèse de la tumeur, ainsi que la non notification systématique des métastases dans certains dossiers; ensuite l'absence des critères histopronostiques en particulier le niveau de CLARCK et de l'indice de BRESLOW sur les résultats anatomopathologiques dans la quasi-totalité des dossiers; et enfin la courte durée de l’étude. Néanmoins cette étude hospitalière donne une orientation sur le mélanome dans notre contexte de travail.

L'incidence hospitalière moyenne était de 4,4 cas par an pour 5000 malades. La majorité des cas était collectée dans le service de chirurgie. Le nombre peu élevé des cas de mélanome dans le service de Dermatologie pourrait être dû au fait que les patients étaient vus tardivement à un stade de lésions ulcérobourgeonnantes, et alors directement orientés dans le service de chirurgie pour une prise en charge chirurgicale qui paraissait évidente à ce stade. Pourtant si les patients étaient vus précocement, la porte d'entrée aurait due être le service de Dermatologie. C'est ainsi que Barro et al. dans une étude portant sur les tumeurs cutanéo-muqueuses menée dans le service de dermatologie du CHU Yalgado Ouédraogo de Ouagadougou, entre le 1er janvier 1992 et le 31 Décembre 1996, ne rapportaient aucun cas de mélanome [5]. Le mélanome semble être un cancer rare chez le sujet noir: Pitché à Lomé au Togo rapportait 63 cas en 20 ans en 2005 [6]. A Bamako au Mali, Diawara rapportait 34 cas en 16 ans en 2008 [7]. Enfin Tossou, un cas en dix ans à Cotonou au Bénin [8], Sène, deux cas en trois ans à Dakar au Sénégal [9] et Kobangué, 22 cas en 10 ans à Bangui en Centrafrique [10]. Comme dans notre série, plusieurs auteurs africains rapportaient un âge moyen au delà de 50 ans voire 60 ans [7, 10–12]; de même qu'une tendance à l’égalisation des sexes [6, 7, 10, 12].

La longue durée d’évolution (3 ans en moyenne) observée chez nos cas était le reflet d'une part de l'ignorance, le patient allant consulté dans un premier temps un tradipraticien, suivi d'un infirmier; et d'autre part de l'inaccessibilité géographique et financière des structures de soins que connaissaient la plus part de ces patients. L'aspect ulcéro- bourgeonnant ainsi que la prédominance de la localisation plantaire étaient aussi rapportés par plusieurs auteurs africains [6, 7, 10–13]. Les localisations des extrémités, notamment des pieds, constituent la particularité du mélanome chez l'Africain: 76 à 91% des cas contre 5 à 10% chez l´Européen [14–16]. Le siège électif du mélanome au niveau des pieds chez l´Africain a fait suggérer dans le passé le rôle des microtraumatismes répétés dus à la marche pieds nus [17]. Mais ce rôle est exagéré, car on observe la même fréquence de localisation des extrémités chez les Noirs Américains [14, 15]. De façon générale les variétés histopathologiques les plus observées chez le noir africain sont le type acrallentigineux et le type nodulaire [7, 10, 12, 18], contrairement aux résultats de Garbe [19] qui notait que le mélanome superficiel extensif (SSM) était le type histopathologique le plus fréquent chez le caucasien. Le diagnostic précoce des cas dans la population, blanche dû aux campagnes de sensibilisations pourrait expliquer cette différence. Même si le niveau de Clarck n'avait pas pu être réalisé chez tous les patients, devant l'aspect clinique des lésions, nous pouvions conclure que la plupart avaient probablement un niveau compris entre III et V comme ceux retrouvés dans les 3 cas où ce niveau a été recherché à l'histopathologie. Ces niveaux de CLARCK élevés traduisant un pronostic péjoratif étaient similaires à ceux retrouvés par Pitche qui rapportait 50 cas dont le niveau de CLARCK était élevé à III et V et Diomandé, chez qui 71% des cas avaient un niveau de CLARCK à IV et V [6, 12]. La présence de métastases dans un nombre élevé de cas nous semblait être en corrélation avec un niveau de Clarck élevé. Dans tous les cas, la taille des lésions et l'aspect nodulaire fréquemment rencontré dans nos cas permettaient dire que le mélanome était une affection de mauvais pronostic dans notre contexte. La prise en charge était essentiellement chirurgicale, la chimiothérapie étant inaccessible financièrement pour la plupart de nos patients.

## Conclusion

Cette étude nous a permis de répondre aux questions que nous nous étions posées et nous pouvions conclure par les observations suivantes: le mélanome dans notre contexte d'exercice était une maladie rare, mais pas exceptionnelle et de révélation tardive, vue souvent au stade locorégional avancé avec des localisations ganglionnaires d'emblée ou des métastases viscérales; il se présentait sous forme de lésions ulcero-bourgeonnantes siégeant préférentiellement au niveau de la région plantaire. Il n'y avait pas de prédominance de sexe car les hommes étaient autant touchés que les femmes et il touchait plus les sujets âgés de plus de 50 ans; le type histopathologique le plus fréquent était type acrallentigineux; les comptes rendus histologiques n’étaient pas complets; le suivi était difficile, beaucoup de patients étaient perdus de vue (étaient-ils décédés?); la prise en charge était tardive, les patients ne consultant qu'au stade de complication avec un pronostic sombre. Le retard à la consultation semblait être le principal facteur de mauvais pronostic dans notre contexte, expliquant le nombre élevé de lésions ulcéro-bourgeonnantes avec des métastases au moment du diagnostic. Une sensibilisation de la population et du personnel de santé sur les signes précoces de la maladie, dans cette localisation plantaire particulière, permettrait probablement de réduire la mortalité, et la morbidité liée à des interventions chirurgicales plus difficiles à un stade avancé.
